# Cellulose Nanofiber–Alginate Biotemplated Cobalt Composite Multifunctional Aerogels for Energy Storage Electrodes

**DOI:** 10.3390/gels9110893

**Published:** 2023-11-11

**Authors:** Felita W. Zhang, Paul D. Trackey, Vani Verma, Galen T. Mandes, Rosemary L. Calabro, Anthony W. Presot, Claire K. Tsay, Timothy J. Lawton, Alexa S. Zammit, Edward M. Tang, Andrew Q. Nguyen, Kennedy V. Munz, Enoch A. Nagelli, Stephen F. Bartolucci, Joshua A. Maurer, F. John Burpo

**Affiliations:** 1 Department of Chemistry and Life Science, United States Military Academy, West Point, NY 10996, USA; felita.zhang@westpoint.edu (F.W.Z.); paul.trackey@westpoint.edu (P.D.T.); vani.verma.mil@army.mil (V.V.); galen.mandes@westpoint.edu (G.T.M.); rosemary.calabro@westpoint.edu (R.L.C.); anthony.presot@westpoint.edu (A.W.P.); claire.tsay@westpoint.edu (C.K.T.); alexa.zammit@westpoint.edu (A.S.Z.); edward.tang@westpoint.edu (E.M.T.); andrew.nguyen@westpoint.edu (A.Q.N.); kenndey.munz@westpoint.edu (K.V.M.); enoch.nagelli@westpoint.edu (E.A.N.); 2 U.S. Army Combat Capabilities Development Command-Armaments Center, Watervliet Arsenal, NY 12189, USA; stephen.f.bartolucci.civ@army.mil (S.F.B.); joshua.a.maurer4.civ@army.mil (J.A.M.); 3 U.S. Army Combat Capabilities Development Command-Soldier Center, Natick, MA 01760, USA; timothy.j.lawton4.civ@army.mil; 4 Photonics Research Center, United States Military Academy, West Point, NY 10996, USA

**Keywords:** biotemplating, cellulose nanofibers, alginate, cobalt, aerogels, catalysis, pseudocapacitors

## Abstract

Tunable porous composite materials to control metal and metal oxide functionalization, conductivity, pore structure, electrolyte mass transport, mechanical strength, specific surface area, and magneto-responsiveness are critical for a broad range of energy storage, catalysis, and sensing applications. Biotemplated transition metal composite aerogels present a materials approach to address this need. To demonstrate a solution-based synthesis method to develop cobalt and cobalt oxide aerogels for high surface area multifunctional energy storage electrodes, carboxymethyl cellulose nanofibers (CNF) and alginate biopolymers were mixed to form hydrogels to serve as biotemplates for cobalt nanoparticle formation via the chemical reduction of cobalt salt solutions. The CNF–alginate mixture forms a physically entangled, interpenetrating hydrogel, combining the properties of both biopolymers for monolith shape and pore size control and abundant carboxyl groups that bind metal ions to facilitate biotemplating. The CNF–alginate hydrogels were equilibrated in CaCl_2_ and CoCl_2_ salt solutions for hydrogel ionic crosslinking and the prepositioning of transition metal ions, respectively. The salt equilibrated hydrogels were chemically reduced with NaBH_4_, rinsed, solvent exchanged in ethanol, and supercritically dried with CO_2_ to form aerogels with a specific surface area of 228 m^2^/g. The resulting aerogels were pyrolyzed in N_2_ gas and thermally annealed in air to form Co and Co_3_O_4_ porous composite electrodes, respectively. The multifunctional composite aerogel’s mechanical, magnetic, and electrochemical functionality was characterized. The coercivity and specific magnetic saturation of the pyrolyzed aerogels were 312 Oe and 114 emu/g_Co_, respectively. The elastic moduli of the supercritically dried, pyrolyzed, and thermally oxidized aerogels were 0.58, 1.1, and 14.3 MPa, respectively. The electrochemical testing of the pyrolyzed and thermally oxidized aerogels in 1 M KOH resulted in specific capacitances of 650 F/g and 349 F/g, respectively. The rapidly synthesized, low-cost, hydrogel-based synthesis for tunable transition metal multifunctional composite aerogels is envisioned for a wide range of porous metal electrodes to address energy storage, catalysis, and sensing applications.

## 1. Introduction

The chemical and physical characteristics of porous composite materials are promising in the development of electrochemical sensors, magnetic actuators, semiconductors, and, in the field of electrochemical energy storage, as supercapacitors [1,2,3,4,5]. Aerogels are an appealing material due to their light weight and high specific surface areas. However, the monolith shape control, synthesis complexity, and mechanical durability of metal/metal oxide aerogels can present significant challenges and highlights the importance of multifunctional materials that satisfy the numerous and often competing requirements for practical device integration [6].

Supercapacitors, or electrochemical capacitors, have a quick recharging ability, high capacitance density, and long life cycle [7,8]. Compared to conventional capacitors, the capacitance per unit volume or mass for supercapacitors can be more than 20–200 times greater due to their ability to harness two storage mechanisms [9,10,11]. Supercapacitors combine the ability of electrochemical double layer capacitors to store charge through ion adsorption, with that of pseudocapacitors to store charge through quick and reversible surface redox reactions [12]. Pseudocapacitive materials commonly consist of transition metal oxides and conducting polymers, offering a high specific capacitance ranging from 20 to 1272 F/g, along with low resistance, thus allowing for a high specific power [8].

Cobalt-based pseudocapacitors are appealing due to cobalt’s natural abundance in compounds, high energy density, electrochemical capacitance, and oxide phase control via thermal treatment [13,14]. However, it is challenging to design electrode materials with a tunable pore structure to optimize electrolyte diffusion, high surface area conductivity, and mechanical strength [13]. Previous methods to address these issues include synthesizing Co nanoparticles in porous activated carbon and creating Co_3_O_4_ nanowires for energy storage applications such as supercapacitors and fuel cells [15,16]. Co used in pseudocapacitors is typically integrated into electrodes through thin carbon sheets or metal films; however, three-dimensional, shape-controlled electrodes offer a higher surface area, and therefore, an increased volumetric and gravimetric capacitance [14]. The pore structure control of Co nanostructures has been previously demonstrated with hydrothermal synthesis by adjusting the time and calcination temperatures [17]. The drawback of hydrothermal synthesis is the high energy requirement, as materials are subjected to high pressure and heat, thus offering an opportunity for lower cost methods [18]. Cobalt oxide aerogel supercapacitors have also been created through an epoxide synthesis route to provide high specific capacitances of up to 600 F/g; however, the mechanical strength of these aerogels are unknown [19,20].

Biotemplating provides a synthesis approach that incorporates inorganic materials into biological structures, leveraging biotemplate geometry, to produce hierarchical composite materials such as aerogels [21]. Biotemplating offers shape-controlled templates, abundant source material, chemical functionalization, and the ability to form three-dimensional porous systems via hydrogels [22,23,24,25,26]. Common biopolymers used in biotemplating include cellulose nanofibers (CNF), alginate, gelatin, silk fibroin, M13 bacteriophage, and bacterial cellulose [25,26,27,28,29,30]. There is a very wide range of demonstrated applications for biotemplated materials, including catalysts, sensors, adsorbents, nanomedicine, and energy storage [31,32,33,34]. Numerous studies have demonstrated the use of biopolymer hydrogels to biotemplate composite metal nanostructured porous electrodes [25,26,35,36,37].

Cellulose provides a useful biotemplate as an abundant polymer found in the cell wall of plants consisting of β(1→4) d-glucose, and is known for its strength and flexibility [25,38,39]. With nanocrystalline structures, diameters of 5–60 nm, and length of up to several micrometers, carboxyl group functionalization allows pH-controllable metal ion binding [40,41,42,43,44,45]. CNFs also offer biocompatibility, a high elastic modulus, and strong interfacial adhesion, and no or low toxicity [39,46,47]. While CNFs are typically ionically crosslinked with metal ions, covalent crosslinking is achieved with toxic and expensive agents such as glutaraldehyde, 1-ethyl-3-(3-dimethylaminopropyl)carbodiimide, and branched polyethylenimine [25,48,49]. Composite metal and metal compound CNF aerogels have been demonstrated for palladium [25,50], single atom cobalt atoms [51], zinc–cobalt metal organic frameworks [52], and molybdenum disulfide [53].

Alginate, another appealing biotemplate, is a polysaccharide naturally found in the cell wall and extracellular matrix of brown seaweed [54,55]. Consisting of 1,4-linked β-d-mannuronic acid and 1,4 α-l-guluronic acid residues with a carboxyl group, alginate may be ionically crosslinked with CaCl_2_ or other metal ions to create stable hydrogels [56,57,58,59]. Alginate gels have been used to biotemplate BiFeO_3_ [60] and NiAl-layered double hydroxide [61], and to encapsulate graphene oxide–cadmium sulfide [62]. One significant limitation of alginate hydrogels, however, is osmotic swelling and collapse based on solution ion concentration, impairing its ability to maintain gel shape control in various material synthesis conditions [63]. Efforts have been made to combine CNF and alginate into a single composite material to utilize the properties of both polymers for bioprinting, tissue engineering, electroactive inks, and wound dressings [64,65,66,67]. Composite CNF–alginate hydrogels’ improved mechanical properties compared to alginate alone, with an increased elastic modulus ranging from 30 to 150 kPa, results from CNF’s stiffness reducing alginate’s syneresis and increasing osmotic resistance in the swollen state [64,65,68], where alginate provides increased elasticity and mechanical resistance to large deformations [65]. While a number of studies report the use of CNF–alginate composite gels for metal ion recovery [69,70], there are limited reports of their combined use for metal biotemplating [71].

Biotemplating approaches involving Co materials include sol-gel synthesis, fungal biomass templates, diatomite templates, and cotton fiber templates [72,73,74,75,76]. The current Co biotemplating approaches involve hierarchical materials that are highly biocompatible, cost-effective, and nontoxic to produce supercapacitive electrodes and lithium-ion battery anode material [74,75,76]. However, there are only a limited number of studies on CNF and alginate for Co metallization for use as energy storage electrodes [77,78]. Additionally, previous studies have not fully demonstrated the challenging trade-offs between maximizing effective surface area and application functionality without compromising the mechanical strength and structural integrity of these porous electrodes.

In this work, we demonstrate CNF–alginate hydrogel biotemplates for cobalt and cobalt oxide composite aerogels that are mechanically durable, magnetically responsive, and electrochemically active. CNF–alginate composite scaffolds provide an easily crosslinked material with structural stability and shape control. Compared to the previous studies of CNF-only aerogels that lacked a simple crosslinking method and alginate-only aerogels that lacked structural stability, this composite biotemplate approach addresses both concerns [48,49,63]. Cobalt nanoparticles integrated into a CNF–alginate scaffold resulting from chemical reduction, rinsing, and supercritical drying with CO_2_ enables a metallized composite material with a tunable metal and metal oxide phase, mechanical strength, magneto-responsiveness, and electrochemical performance via pyrolysis and thermal oxidation. To build on the work of previous biotemplated aerogels, this synthesis approach demonstrates the trade-offs between the material’s properties and the potential electrochemical and magnetic application performance. Compared to monodisperse nanoparticles, solvothermal syntheses, and thermal decomposition approaches for cobalt oxides, the material phase control, mechanical strength, surface area, conductivity, small particle size, and high energy densities of the CNF–alginate–Co aerogels offer a tunable synthesis approach for mechanically robust, multifunctional biotemplated aerogels for a wide range of applications.

## 2. Results and Discussion

### 2.1. CNF–Alginate–Cobalt Aerogel Synthesis

**Figure 1:**
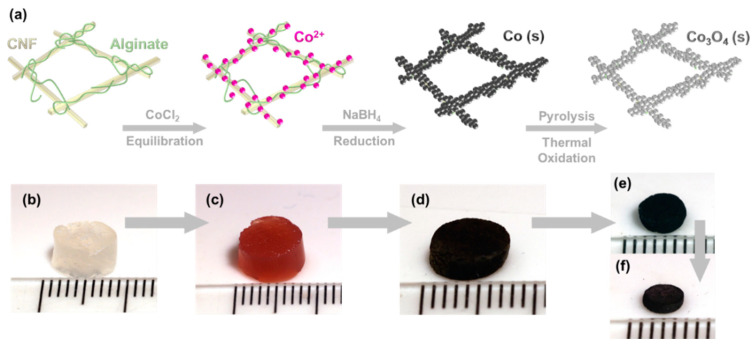
(**a**) Aerogel synthesis scheme. (**b**) A solution of CNF and sodium alginate is physically crosslinked through mixing and centrifugation. (**c**) CNF–alginate precursor solution (1:1, *w*/*w*) equilibrated in 2 M salt solution consisting of a 1:1 ratio of CaCl_2_ and CoCl_2_, creating an ionically crosslinked hydrogel. (**d**) CNF–alginate–cobalt after reduction with NaBH_4_, rinsing, solvent exchange with ethanol, and supercritical drying. (**e**) Aerogel from (**d**) pyrolyzed in N_2_(g) at 550 °C for 1 h. (**f**) Aerogel from (**e**) thermally oxidized in air at 550 °C for 1 h. Ruler scale in (**b**–**f**) is 1 mm between tick marks.

Figure 1a depicts the synthesis scheme for the CNF–alginate–cobalt aerogels with pyrolysis and thermal annealing to control the inorganic material phase and multifunctional properties. The physically entangled CNF–alginate hydrogels (1:1, 3% *w*/*w*) (Figure 1b) were equilibrated in a 2 M CoCl_2_–CaCl_2_ solution (Co^2+^:Ca^2+^ at 1:1), as shown in Figure 1c. The relatively high concentration of the salt solution was necessary to create a sufficiently high concentration gradient to drive the diffusion of ions into the gel matrix [79,80]. The divalent calcium ions served as ionic crosslinkers between the carboxylate groups on both the CNF and alginate biopolymers. The cobalt ions were electrostatically bound to the anionic carboxylates throughout the organic structure and were also sequestered within the gel pores, where they were prepositioned for chemical reduction. High-concentration 2 M NaBH_4_ was used for the diffusion of the reducing agent into the gel, especially given that its concentration is depleted with the reduction of Co^2+^ ions, and to ensure a complete and uniform reduction throughout the gel volume [81,82,83,84]. The chemical reduction of the Co^2+^ ions into cobalt metal changed the appearance of the gels from a pink hue to a black gel, as seen in Figure 1d. Cutting through the resulting gel cross-section revealed a uniform black color throughout the gel, indicating complete diffusion and chemical reduction. The samples will be referred to as supercritically dried, pyrolyzed, and thermally oxidized for the discussion that follows.

In order to preserve the pore structure of the gel, the gels were solvent exchanged in ethanol and supercritically dried. The resulting aerogel seen in Figure 1d shows macroscale shape retention when compared to the wet gel before drying and the black color indicates the presence of cobalt metal. To increase the electrical conductivity of the material, the supercritically dried samples were then pyrolyzed in N_2_(g) at 550 °C for 1 h (Figure 1e), and to convert the cobalt metal to Co_3_O_4_, the aerogels were thermally oxidized by placing them in the furnace at 550 °C for 1 h under ambient air, as shown in Figure 1f. Volume shrinkage occurs when a supercritically dried CNF–alginate–Co aerogel is pyrolyzed, as the carbon compounds and biomass from the CNF and alginate are volatilized. The average volume of the supercritically dried samples was 0.156 ± 0.023 cm^3^ and the average volume of the pyrolyzed aerogel was 0.0448 ± 0.0119 cm^3^, indicating a shrinkage of approximately 70% by volume. The average volume of the thermally oxidized samples was 0.0416 ± 0.00720 cm^3^. The volume decrease from the pyrolyzed to thermally oxidized sample was 7%. The resulting sample densities of the supercritically dried, pyrolyzed, and thermally oxidized aerogels were 0.16 ± 0.2, 0.27 ± 0.08, and 0.24 ± 0.3 g/cm^3^, respectively. Pyrolyzing the supercritically dried aerogels results in a 1.7-fold increase in density, while thermal oxidation slightly decreases the sample density, likely due to the oxidation of the residual carbon and the lower density of the Co_3_O_4_ phase compared to metallic cobalt.

### 2.2. Fourier Transform Infrared Spectroscopy (FTIR)

**Figure 2:**
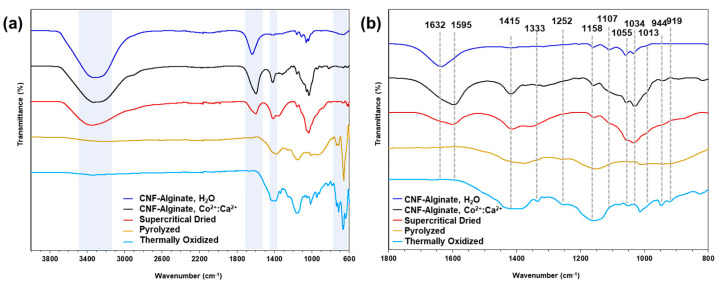
(**a**) FTIR spectra from 4000–600 cm^−1^. (**b**) Spectra from (**a**) for 1800–800 cm^−1^; numbered peak positions have units of cm^−1^.

To determine the influence of the composite aerogel synthesis method on the biotemplate hydrogel scaffold, FTIR was conducted at the various stages of synthesis and is shown in Figure 2. As seen in the survey spectrum in Figure 2a, the broad peak centered at approximately 3300 cm^−^^1^ represents the -OH stretching present on both CNFs and alginate and shifts slightly higher to 3350 cm^−^^1^ in the presence of a 2 M solution of 1:1 Co^2+^:Ca^2+^ ions. As expected, this broad -OH peak disappears with pyrolysis and the carbonization of the composite aerogel organic phase. As shown in Figure 2b, the well-defined peak at 1632 cm^−^^1^ is indicative of -COO^−^ asymmetric stretching, present in the carboxyl groups of both the CNFs and alginate. The -COO^−^ peak shifts down to 1595 cm^−^^1^ in the presence of a 2 M solution of 1:1 Co^2+^:Ca^2+^, when the carboxyl group complexes as a salt, and vanishes after the pyrolysis of the organic phase. The downshift of the -COO^−^peak is likely due to the -COO^−^ bond weakening after donating electrons from the bond to metal ions [85]. The disappearance of the peak following pyrolysis is due to the bonds in the functional group being broken at a high energy and temperature [86]. The symmetric stretching of the carboxylic acid salt is also observed in a small peak at 1415 cm^−^^1^, which appears with the addition of 1:1 Co^2+^:Ca^2+^ and vanishes after chemical reduction. The -CO stretching at 1107 cm^−^^1^ corresponds to the alkyl-substituted ethers, present between the saccharide subunits of both CNFs and alginate, and present at the carboxymethyl substitution on the CNFs. This peak remains present until carbonization. The abundance of secondary alcohols is observed in the peak at 1055 cm^−^^1^, representing -CO stretching, and also disappears after pyrolysis. The -CO stretching of primary alcohols can be observed at the 1034 cm^−^^1^ peak prior to pyrolysis.

### 2.3. Scanning Electron Microscopy (SEM)

**Figure 3:**
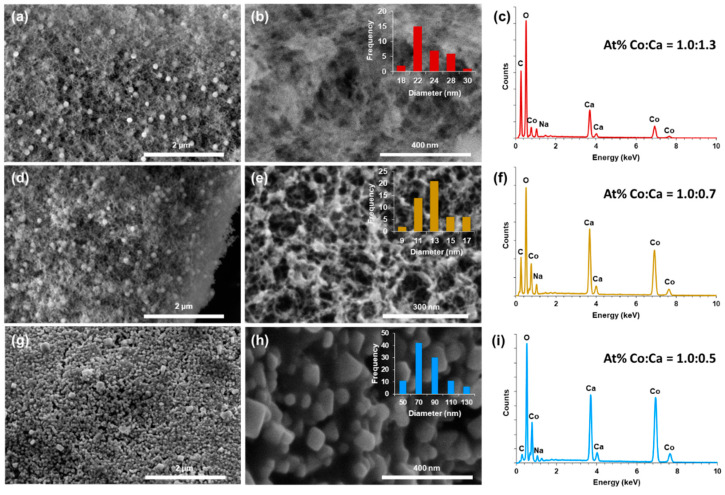
SEM images and EDS of (**a**–**c**) supercritically dried CNF–alginate–Co aerogels. (**d**–**f**) Aerogel from (**a**) pyrolyzed in N_2_(g) at 550 °C for 1 h. (**g**–**i**) Aerogel from (**d**) thermally oxidized in air at 550 °C for 1 h.

The structure of the nanoparticles and nanofibrils in the SEM images in Figure 3 show a high surface area porous network, and the EDS spectra demonstrate the successful metallization with cobalt after chemical reduction. The supercritically dried aerogels (Figure 3a,b) have fiber diameters ranging from 18 to 30 nm with an average of 22 ± 3 nm. Following pyrolysis in nitrogen gas, the fiber diameter decreased to a range of 9 to 17 nm with an average of 12 ± 2 nm (Figure 3e) and is attributed to the carbonization of the biopolymer scaffold. The pyrolyzed samples were then thermally oxidized to allow for the formation of Co_3_O_4_ for pseudocapacitor applications [87]. Following thermal oxidation (Figure 3g,h), material aggregation into large particles ranging from 50 to 130 nm with an average size of 77 ± 22 nm was seen, replacing the nanofibril network as the predominant structure. The larger bead-like spherical structures seen in Figure 3a,b,d,e exhibit a structure distinctly different to the three-dimensional wire-like network overall and are believed to be composite aggregates of alginate and cobalt/cobalt oxide. The spherical surfaces present a fibril-like texture, and the supercritical and pyrolyzed spheres have an average diameter of 267 ± 53 nm and 140 ± 22 nm, respectively, corresponding to a 48% decrease (Figure S1).

The EDS spectra for the supercritically dried samples (Figure 3c) indicate a Co to Ca atomic ratio of 1.0:1.3, consistent with the CoCl_2_–CaCl_2_ solution at a 1:1 ratio that the CNF–alginate hydrogels were equilibrated in. Following pyrolysis, the EDS spectrum in Figure 3f indicates a decrease in the relative amount of Ca, with a Co to Ca ratio of 1.0:0.7. The decrease in Ca is likely due to the decomposition of the ionically crosslinked biopolymer scaffold, as alginate crosslinked with CaCl_2_ begins to significantly decompose above 250 °C with the loss of Ca^2+^ ions occurring between 150 and 400 °C due to the carbonization of alginate and the loss of hydroxyl and carboxyl groups [88,89]. With the subsequent thermal oxidation, the Co:Ca ratio became 1.0:0.5. The further decrease in the relative balance of Ca is likely due to the further decomposition of the carbonized scaffold.

### 2.4. X-ray Diffractometry (XRD)

**Figure 4:**
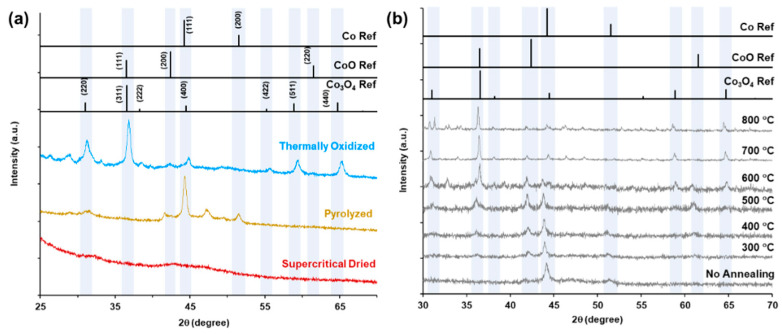
X-ray diffraction patterns. (**a**) Supercritically dried, pyrolyzed, and thermally oxidized CNF–alginate–Co aerogels. (**b**) Pyrolyzed CNF–alginate–Co aerogels heated in increments of 100 °C from ambient to 800 °C. Indexed JCPDS files for Co, CoO, and Co_3_O_4_ are 01-077-7451, 01-083-4544, and 01-080-1543, respectively.

The XRD patterns for the supercritically dried, pyrolyzed, and thermally oxidized samples are shown in Figure 4a. The supercritically dried sample does not exhibit any distinct peaks, with a very broad, low intensity peak centered at approximately 43°, which is close to the (111) peak of cubic cobalt (JCPDS 01-077-7451) at 44.2°. The clear presence of cobalt in the EDS spectra with the lack of distinct XRD peaks and low-intensity broad cobalt peaks suggests very small crystallite sizes or the possibility of amorphous cobalt [90,91].

The pyrolyzed sample peaks at 44.2° and 51.5° index to the (111) and (200) peaks of cobalt with a cubic crystal system (JCPDS 01-077-7451). An additional pyrolyzed sample peak at 47.2° indexes to hexagonal cobalt (JCPDS 01-071-4652) suggesting a mixed cubic and hexagonal crystal system for the pyrolyzed sample cobalt phase. To determine the ability to thermally oxidize the cobalt phase to Co_3_O_4_, the pyrolyzed samples were thermally annealed in air from ambient temperature to 800 °C and scanned every 100 °C, starting at 300 °C (Figure 4b). At 400 °C, peaks at 36.2 ° and 61.3 ° emerge, indexing to CoO (111) and (200) for JCPDS 01-083-4544, as well as the (311) peak for Co_3_O_4_ for JCPDS 01-080-1543. At 800 °C, the Co_3_O_4_ phase peaks have the highest intensity with a phase composition of 83% Co_3_O_4_ and 17% CoO. The structure of the thermally oxidized samples is shown in the SEM micrographs in Figure S2. Thermal oxidation at 550 °C in air for 1 h was selected as the optimal temperature to maximize the Co_3_O_4_ phase and minimize the particle aggregation and pore network volume decrease.

### 2.5. X-ray Photoelectron Spectroscopy (XPS)

XPS was carried out on the sample after each reaction step to confirm the chemical composition. The preliminary survey scans showed the initial sample, following chemical reduction, rinsing, and supercritical drying, was composed of mostly oxygen and carbon with cobalt present (Figure S4). Sodium and boron were also detected and likely originate from the reduction with sodium borohydride. Calcium was also detected originating from its use for ionic crosslinking in the composite hydrogel preparation.

**Figure 5:**
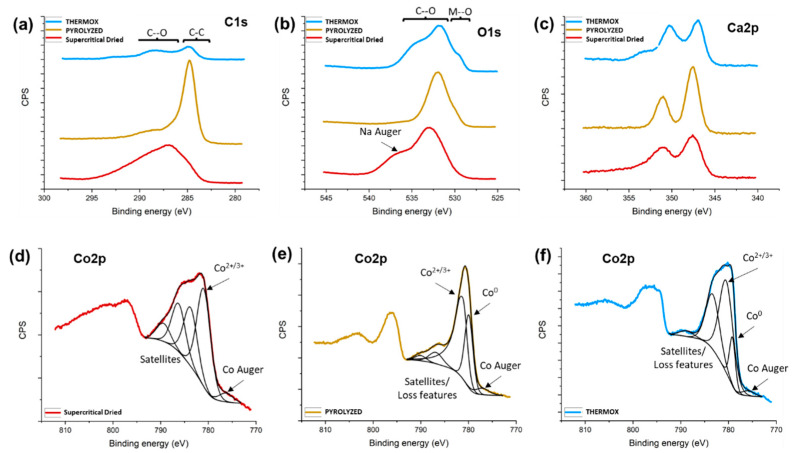
XPS high resolution scans of (**a**) C1s, (**b**) O1s, (**c**) Ca2p, (**d**) Co2p for supercritically dried aerogels, (**e**) Co2p for pyrolyzed aerogels, and (**f**) Co2p for thermally oxidized aerogels. (**d**–**f**) Deconvoluted spectra for the Co2p region for each sample are shown with black lines.

To gain insight into the chemical species present at the surface at each reaction step, higher resolution scans were collected for the cobalt, carbon, oxygen, and calcium regions (Figure 5). From the carbon 1s region in Figure 5a, the supercritically dried sample shows a broad peak spanning 285–295 eV, indicating several C-C and C-O groups are present on the surface. Once the sample is pyrolyzed, there is a significant decrease in the relative intensity of the peaks, corresponding to C-O groups, and an increase in the relative peak intensity, corresponding to C-C sp^3^ groups (284.8 eV). Following oxidation, there is an overall decrease in intensity in the C1s region indicating carbon-containing species have been removed from the surface.

In the O1s region (Figure 5b), care must be taken when interpreting the spectra since Na was detected in the survey scans and a Na Auger peak overlaps with a portion of the O1s region (535 eV). The Na Auger peak was present in all three samples. When pyrolyzing the sample, there is a shift in intensity from 533 eV to 531–532 eV which, like the C1s scans, suggests a more uniform sample compared to the initial state. After oxidizing the sample, a shoulder emerges at 529–530 eV, consistent with metal oxide species. Figure 5c indicates that calcium, used as an ionic crosslinker in the hydrogel biotemplate, is present in the supercritically dried, pyrolyzed, and thermally oxidized samples.

The Co2p region can be difficult to interpret compared to other elements, owing to the multiple oxide species that can be present, the proximity of the XPS peaks with satellite peaks, the loss features and Auger peaks, and that the Co^2+^ and Co^3+^ XPS peaks exactly overlap [92,93]. The deconvoluted spectra for the Co2p region for each sample are shown in Figure 5d–f. The supercritically dried sample in Figure 5d shows it is predominantly oxidized Co at the surface, with a peak at 781 eV, consistent with Co^2+/3+^. This contrasts with the XRD results showing the sample is mostly metallic Co. It is known that Co samples can show differences in chemical composition between XPS and XRD [94]. This could arise due to the reactive nature of Co and the surface readily oxidizing in the atmosphere to form an oxidized surface layer above metallic Co in the bulk. Other peaks in the spectrum correspond to Co Auger (776 eV) and satellite features (784–790 eV). Further discrimination between Co^2+^ and Co^3+^ is not possible since the XPS peaks overlap and, if Co_3_O_4_ is present, it is a mixed valence of 2+ and 3+ oxidation states [93]. Following pyrolysis, a metallic Co peak is detected at 780 eV in addition to the oxide peak at 781 eV in a 1:2 ratio, respectively. The scans of the oxidized sample reveal that the ratio of metallic to oxidized Co decreases to 1:4, confirming an increase in the Co surface oxidation.

### 2.6. Thermogravimetric Analysis (TGA)

**Figure 6:**
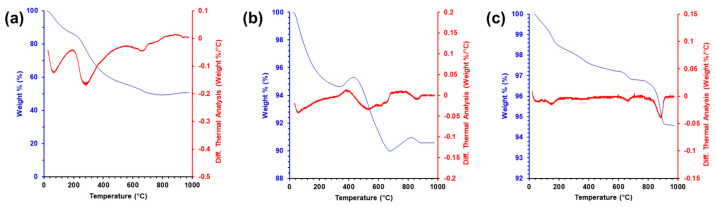
TGA and DTA for (**a**) supercritically dried, (**b**) pyrolyzed, and (**c**) thermally oxidized aerogels.

The TGA and differential thermal analysis (DTA) were conducted on the supercritically dried, pyrolyzed, and thermally oxidized aerogels shown in Figure 6. The supercritically dried gels lost around 49.3% of their initial mass due to the loss of organic matter from both the CNFs and sodium alginate. Figure 6a supports the analysis with the first major DTA peak around 100 °C, consistent with the dehydration of alginate [95], and the second DTA peak, beginning at around 280 °C, which is consistent with the observed temperature for the thermal degradation of CNFs, as well as the degradation of the glycosidic bonds in the alginate [96]. From this, it is estimated that the supercritically dried gels have a 1:1 (*w*/*w*) ratio of inorganic to organic mass. The TGA data for the pyrolyzed sample show an immediate decrease in mass followed by a slight increase, consistent with a drying and buoyancy effect that has been observed previously in silica aerogels [97,98]. The second DTA peak in Figure 6b is consistent with the conversion of alginate to sodium carbonate [95]. The thermally oxidized sample in Figure 6c displays only one prominent DTA peak around 950 °C. This is consistent with the decomposition of Co_3_O_4_ into CoO and oxygen gas that occurs around that temperature [99]. In both the pyrolyzed and thermally oxidized samples, the total mass loss is less than 9% of the sample, indicating an over 9:1 ratio of inorganic to organic matter.

### 2.7. Nitrogen Gas Adsorption–Desorption

**Figure 7:**
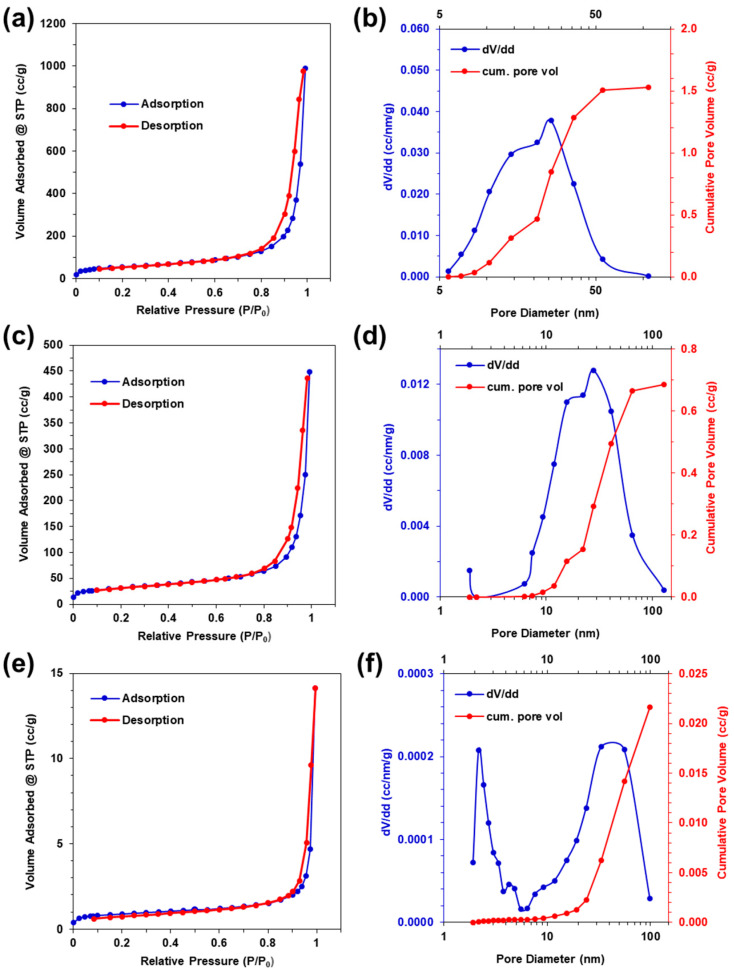
Nitrogen adsorption–desorption isotherms, and pore size distribution with cumulative pore volume for (**a**,**b**) supercritically dried aerogels, (**c**,**d**) pyrolyzed aerogels, and (**e**,**f**) thermally oxidized aerogels.

Nitrogen gas adsorption–desorption was performed to characterize the aerogel surface area and pore size distribution. The isotherms, pore size distribution, and cumulative pore volumes for the supercritically dried, pyrolyzed, and thermally oxidized aerogels are shown in Figure 7. All three aerogel types exhibit mixed type II and type IV isotherms, with type III hysteresis, according to IUPAC classification standards, indicating the presence of both meso- and macropores [100]. A slight rise in the adsorbed volume is seen at low relative pressures of P/P_o_ less than 0.1, with no limiting adsorption at high relative pressures greater than 0.9, indicating the presence of 2–50 nm mesopores and greater than 50 nm macropores [101]. At the maximum relative pressure of P/P_o_ = 0.995, the supercritically dried, pyrolyzed, and thermally oxidized aerogels have decreasing maximum adsorbed volumes of 989, 448, and 14 cm^3^/g, respectively; and the BET specific surface areas (SSA) are 190, 111, and 3.4 m^2^/g, respectively. This indicates that pyrolyzing the supercritically dried samples results in a 55% decrease in the adsorbed volume and a 42% decrease in the BET specific surface area. The subsequent thermal oxidation of the pyrolyzed samples results in a further decrease of 97% for both the adsorbed volume and BET specific surface area. The large decrease in the volume and area upon thermal oxidation are attributed to the coalescence of mass into large particle aggregates, as seen in Figure 3g,h.

The BJH analysis (Table 1) of the supercritically dried, pyrolyzed, and thermally oxidized desorption isotherms results in cumulative pore volumes of 1.53, 0.69, and 0.02 cm^3^/g; specific surface areas of 228, 93, and 2.7 m^2^/g; and average pore sizes of 26.9, 30.0, and 22.8 nm, respectively [102]. The supercritically dried sample specific surface area is comparable to palladium metallized CNF and silk aerogels [25,30]. The pyrolysis of supercritically dried samples results in 55% and 59% decreases in the cumulative pore volumes and specific surface areas, respectively, with subsequent thermal oxidation resulting in a further decrease of 97% of both the adsorbed volume and specific surface area, indicating a large trade-off between the pore volume and surface area, with the tuning of inorganic phase composition.

### 2.8. Mechanical Characterization

**Figure 8:**
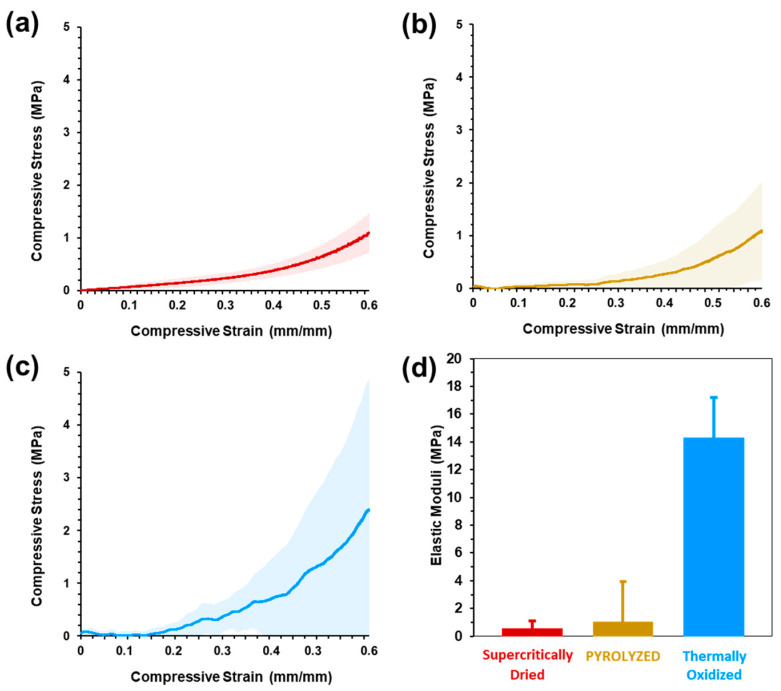
Aerogel compressive stress–strain curves for (**a**) supercritically dried, (**b**) pyrolyzed, and (**c**) thermally oxidized aerogels (*n* = 5 for each sample type; solid line represents the average stress with standard error of the mean in the shaded regions). (**d**) Elastic moduli.

Figure 8a–c shows the compressive stress–strain curves of the three sample types, where the solid line represents the average data (*n* = 5) and the shaded regions depict one standard deviation above and below the mean. The representative compressive stress–strain curves are shown in Figure S5. The supercritically dried, pyrolyzed, and thermally oxidized aerogels all exhibited a narrow elastic region less than 0.1 strain. For the supercritically dried sample, a gradual increase in the slope during plastic deformation occurs between 0.1 and 0.4 strain before material densification is seen above 0.4 strain. While the standard deviation of the supercritically dried samples gradually increases with increasing strain, the pyrolyzed and thermally oxidized samples exhibit a greater variability and brittle material behavior through the full strain range. The pyrolyzed and thermally oxidized samples showed an extremely limited elastic range with less than 0.01 strain, before successive fracture with increasing strain (Figure S5). The brittle behavior under compressive loads is attributed to the cleavage of the biopolymer glycosidic bonds and the corresponding loss of the hydrogen bonding of the CNF–alginate biotemplate upon pyrolysis [103] and conversion of the cobalt metallic phase to predominantly Co_3_O_4_ metal oxide, as seen in XRD patterns (Figure 4b), upon thermal oxidation [104,105].

The elastic moduli were calculated from the slope of the elastic regions of the compressive stress–strain data and are shown in Figure 8d. The supercritically dried, pyrolyzed, and thermally oxidized samples had elastic moduli of 0.58 ± 0.54, 1.1 ± 2.8, and 14.3 ± 2.9 MPa, respectively. The elastic moduli of all the supercritically dried and pyrolyzed samples exhibit large standard deviations relative to the mean moduli and are attributed to the very limited elastic response strain range. While the thermally oxidized samples had the highest average elastic modulus, it also had the smallest elastic range before plastic deformation or initial fracture. The significant increase in the elastic moduli of the thermally oxidized samples is likely attributable to the increased material density, decreased macropore volume (Figure 7f), and formation of large aggregates seen in SEM imaging (Figure 3g,h) compared to supercritically dried aerogels. Studies of supercritically dried versus thermally oxidized silica and graphene aerogels have shown that heating causes increased network connectivity and the collapsing of pores to increase the bulk density, thus making the material more resistant to deformation [106,107]. The supercritically dried average elastic modulus is comparable to that of CNF-only aerogels, ranging from 56 kPa to 5.310 MPa [108] and is comparable to an analogous CNF–boron nitride nanosheet aerogel with 0.335 MPa [100].

### 2.9. Vibrating Sample Magnetometry (VSM)

**Figure 9:**
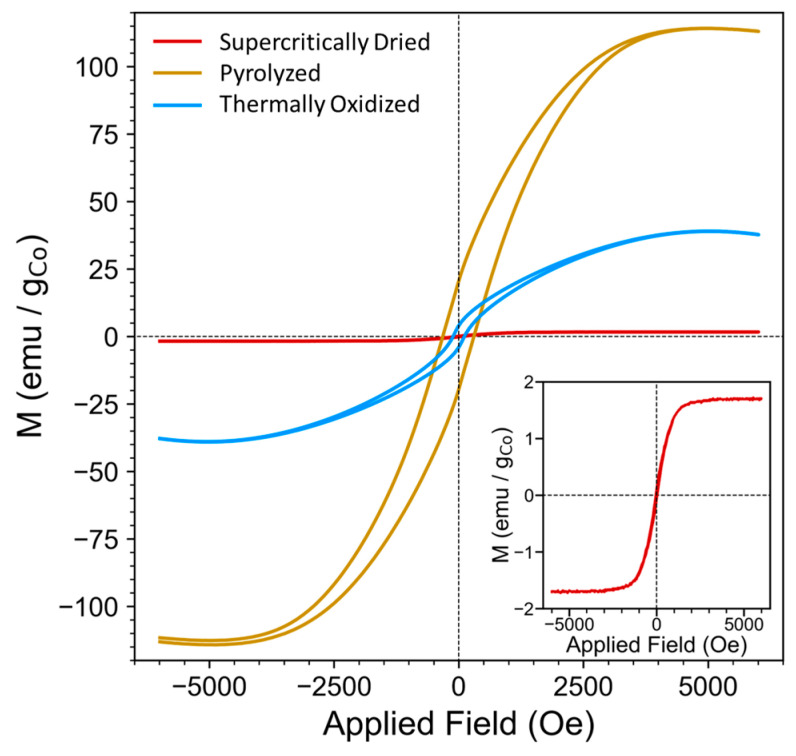
VSM hysteresis curves of the supercritically dried, pyrolyzed, and thermally oxidized aerogels. The inset shows a zoomed-in plot of the supercritically dried sample. The hysteresis curves were normalized for mass of cobalt in each sample as determined from ICP measurements.

The magnetic measurements show that the various processing steps allow for the tuning of the aerogel magnetic properties. The supercritically dried, pyrolyzed, and thermally oxidized aerogels were analyzed by VSM to understand how the various processing steps influenced their magnetic properties (Figure 9). The hysteresis curves for the samples were measured in the scan range of −6000 Oe to 6000 Oe, and their magnetizations were normalized per mass of cobalt content for each sample, as determined from the ICP-OES (Figure S3, Table S1). Prior to pyrolysis or thermal annealing, the sample shows very low saturation magnetizations (M_s_), remnant magnetization (M_r_), as well as a narrow coercivity (H_c_), as summarized in Table 2.

After pyrolysis, the aerogels have harder magnetic properties, as indicated by their increase in remanence and coercivity [109]. The saturation also increases, which can be attributed to the changing structure of the cobalt particles within the aerogels, since M_s_ is known to be influenced by both the size and crystallinity of the magnetic domains [110,111]. The SEM and XRD show that the overall ligament feature size decreased, while the crystallinity of the cobalt phase increased following pyrolysis, which can account for the increase in the observed M_s_, M_r_, and Hc and demonstrates the tunability of the magnetic properties through thermal treatment. The thermal oxidation of the aerogels further converted them to Co_3_O_4_ which is antiferromagnetic in the bulk phase [112]. Bulk Co_3_O_4_ has a normal spinel structure where oxygen anions have a cubic close-packed structure and Co^2+^ and Co^3+^ cations occupy tetrahedral and octahedral interstitial sites, respectively. The Co^3+^ is 3d^6^ and low spin so all of its electrons are paired [112,113]. Thus, all of the unpaired electrons that are present in Co_3_O_4_ are in Co^2+^ tetrahedral sites, and these sites are surrounded by the four nearest neighbors with opposite spins, which leads to the observed antiferromagnetic behavior [112,114]. However, when Co_3_O_4_ is converted from bulk to nanoscale, factors such as uncompensated surface spins, ion exchange, and oxygen vacancies can cause a partial conversion to an inverse spinel, allowing for some ferromagnetic properties [113,115]. The hysteresis curve for the thermally annealed sample clearly shows some ferromagnetic behavior, with a M_r_ and Hc greater than zero [116]. The shape of the thermally oxidized hysteresis curve also shows a slight perminvar shape, which has been reported for some cobalt-containing magnetic materials [111]. The magnetic properties, including M_s_, M_r_, Hc, and the hysteresis curve shape, could all be influenced by factors including the heating rate, cooling rate, presence of any impurities, furnace atmosphere, crystal structure, and exposed crystal facets [110,111,114], and further studies are needed to fully understand the influence of the thermal oxidation of the magnetic properties of the aerogels. However, the results presented here show the versatility and tunability of the magnetic properties, thus demonstrating the multifunctionality of the aerogels.

### 2.10. Electrochemical Characterization

**Figure 10:**
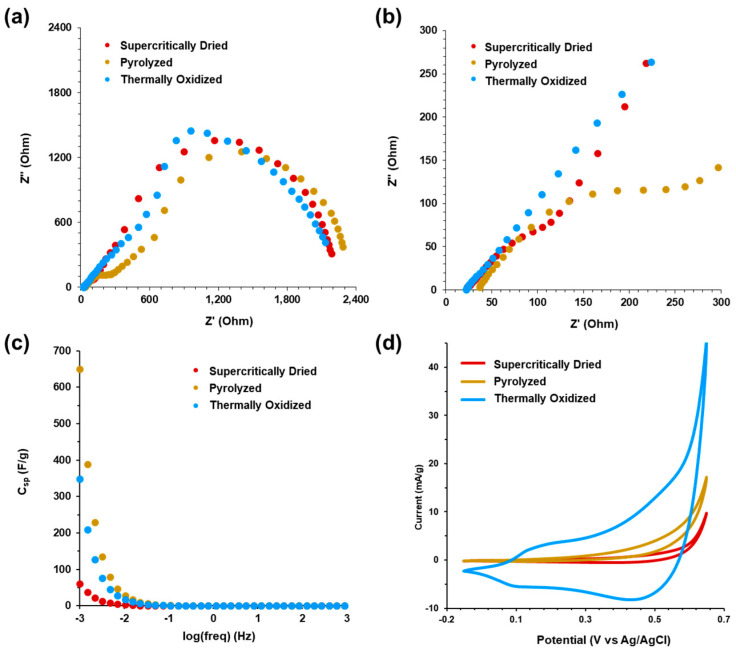
Electrochemical characterization in 1M KOH of supercritically dried, pyrolyzed, and thermally oxidized aerogels. (**a**) Electrochemical impedance spectroscopy (EIS) from 100 kHz to 1 mHz. (**b**) High frequency impedance from (**a**). (**c**) Specific capacitance (C_sp_) vs. log frequency from (**a**). (**d**) Cyclic voltammetry at scan rate of 1 mV/s. Data in (**a**−**d**) are plotted individually in Figure S6.

Electrochemical impedance spectroscopy (EIS) and cyclic voltammetry (CV) was performed in 1.0 M KOH for the supercritically dried, pyrolyzed, and thermally oxidized aerogels, and the results are shown in Figure 10 (data in Figure 10a–d are plotted individually in Figure S6). EIS was performed in a frequency range of 10^5^ to 10^−^^3^ Hz to generate the Nyquist plots, shown in Figure 10a. The high frequency region from 10^5^ Hz to 200 Hz is shown in Figure 10b. For the supercritically dried, pyrolyzed, and the thermally oxidized aerogels, there was no distinct difference in the high frequency resistance intercept with the real (Z’) axis, which includes the total ohmic resistance, the ionic solution resistance, and the charge transfer resistance at the electrolyte interface [117]. The high frequency resistance intercepts for the supercritically dried, pyrolyzed, and thermally oxidized aerogels were 23.2 Ω, 36.6 Ω, and 22.4 Ω. However, the supercritically dried aerogel high frequency range shows a partial depressed semi-circle, which is larger and more distinct for the pyrolyzed sample, and it is an approximately 45° line for the thermally oxidized aerogels, indicating a low charge transfer resistance for electrolyte diffusion into the porous aerogel network [117,118]. The high frequency resistance of the thermally oxidized sample decreased compared to both the supercritically dried and pyrolyzed aerogels and, as shown in the SEM images in Figure 3g,h, this decrease can be attributed to the observed coalescence of the large aggregates within the aerogel network leading to improved conductivity. The low frequency region of the EIS spectra for the supercritically dried, pyrolyzed, and thermally oxidized aerogels shows an incomplete semi-circle converging to the real Z’ (Ohm) axis from the restrictive mass transfer of electrolytes within the network of the structure due to the pore size distribution of the CNF–alginate biotemplate with the presence of Co/Co_3_O_4_ particle aggregates. The general porous structure and the large semi-circle in the low frequency region results from the charge transfer resistance for diffusion through the meso- and microporous network. Figure 10c shows the specific capacitance in Farads per gram versus the log of frequency. At 1 mHz, the specific capacitances of the supercritically dried, pyrolyzed, and thermally oxidized aerogels were 61 F/g, 650 F/g, and 349 F/g, respectively, based on the total sample mass. When adjusted for the active cobalt and cobalt oxide mass in the samples, based on the TGA results from Figure 6, the specific capacitances are estimated to be 122 F/g, 722 F/g, and 387 F/g, respectively. Overall, pyrolysis and thermal oxidation increased the specific capacitance of the CNF–alginate–Co supercritically dried aerogels, resulting in values similar to epoxide-based cobalt oxide aerogel supercapacitors [19,20].

The cyclic voltammetry of the aerogels from −0.1 V (vs. Ag/AgCl) to +0.6 V (vs. Ag/AgCl) at a 1 mV/s scan rate is shown in Figure 10d (CV scans for the supercritically dried, pyrolyzed, and thermally oxidized aerogels at 50, 25, 10, 5, and 1 mV/s are shown in Figure S7). The redox peaks for the supercritically dried and pyrolyzed samples are less distinct than the thermally oxidized aerogel, in which a significant increase in the specific current density is observed across the voltage range. For the thermally oxidized samples with predominant Co_3_O_4_, as seen in the XRD data (Figure 4), the first redox couple observed in the +0.1 V to +0.2 V (vs. Ag/AgCl) potential range corresponds to the oxidation of Co^2+^ to Co^3+^ and subsequent reduction of Co^3+^ to Co^2+^ [119,120]. The prominent redox couple peaks within the potential range of +0.4 V to +0.6 V (vs. Ag/AgCl) is attributed to the formation of cobalt oxide (CoO_2_) and the adsorption of OH^−^ ions from reversible faradaic reaction of Co^3+^ to Co^4+^ on the aerogel surface [119,120].

### 2.11. Aerogel Comparison

Table 3 summarizes the material characterization values for the supercritically dried, pyrolyzed, and thermally oxidized samples to enable direct comparisons. Subsequent to initial synthesis and supercritical drying, the pyrolysis, and thermal oxidation treatments significantly change the predominant cobalt material phase, nanostructure diameter, pore sizes, and surface area, along with the elastic modulus, magneto-responsiveness, and electrochemical capacitance. The three sample types and synthesis methods enable tuning each respective property over an appreciable range of values. However, as expected, the increase in some material properties comes with a concomitant decrease in others, changing their potential applications. For the supercritically dried samples, the very small crystallite size or small amorphous presence of cobalt, along with meso- to macro-porosity with a robust mechanical response, suggests the possibility for cobalt nanoparticle catalytic applications in practical devices [121]. While further testing is required to determine specific reaction catalysis and kinetics, the supercritically dried aerogel’s high specific surface area and mechanical durability during physical manipulation with large compressive strain range before densification make it a useful platform for transition metal applications amenable to the direct chemical reduction synthesis.

In general, pyrolysis decreased the nanostructure ligament diameter, calcium content, and surface area, but increased the average pore diameter, saturation magnetization, remnant magnetization, and coercivity, as well as increasing the elastic modulus and electrochemical capacitance. Pyrolysis also made the presence of crystalline cobalt more distinct in the XRD patterns compared to the supercritically dried samples, indicating larger crystallite sizes. The pyrolyzed samples exhibited the highest magneto-responsiveness and electrochemical responsiveness of all three sample types, suggesting their use in magnetic sensing and actuation, as well as for capacitive energy storage devices [14]. However, the limited elastic strain range in compression and brittle material response may limit applications involving internal device strain and large vibrational loads. The thermal annealing of the pyrolyzed aerogels shifted the predominant cobalt phase to Co_3_O_4_ and increased the elastic modulus to the highest value of 14.3 ± 2.9 MPa, though with a very limited elastic strain range, resulting in a brittle material compared to the supercritically dried sample. The thermally annealed aerogels provide the highest capacitance determined by cyclic voltammetry, demonstrating the benefit and tunability by the thermal annealing of metals to metal oxides, which should be useful for numerous transition metals.

To further demonstrate the tunability of the CNF–alginate hydrogel biotemplate with metallization via chemical reduction, with pyrolysis and thermal annealing as post synthesis treatments, multiple approaches are envisioned. First, changing the relative composition of CNF to alginate, as well as the overall biopolymer hydrogel weight percentage may enable the tuning of the pore size distribution and mechanical properties of the resulting supercritically dried aerogel. Controlling the transition metal ion concentration, and its ratio with Ca^2+^ ions as ionic crosslinkers should enable the tuning of the ratio of metal to organic template, as well as mediating multi-metallic or alloy compositions though hydrogel equilibration with mixed ion species solutions. This mixed ion species approach might also be used to tune magneto-responsiveness through combinations of cobalt, nickel, and iron ferromagnetic ions. Different combinations of metal ions and subsequent thermal oxidation might be used to achieve a range of mixed metal oxide materials for catalytic and energy storage applications.

## 3. Conclusions

In this work, we have demonstrated the synthesis of CNF–alginate biotemplated cobalt aerogels with tunable inorganic phase composition, magneto-responsiveness, elastic modulus, and charge storage through pyrolysis and thermal oxidation. The chemical reduction of cobalt ions within the CNF–alginate hydrogel scaffold followed by supercritical drying resulted in aerogels with high specific surface area and meso- to macroporous networks. Pyrolysis and thermal oxidation greatly increased magnetic response and electrochemical capacitance, but with the tradeoff of decreasing monolith and pore volumes and increasing material brittleness. The composite CNF–alginate hydrogel biotemplating method to achieve metal and metal oxide aerogels offers a tunable approach for multifunctional materials through modifying material composition, pore size, and mechanical properties, resulting in differences in the magnetic and electrochemical performance. Future work varying the composite hydrogel template, the range of transition metals, to include the co-reduction of multiple ions, and post synthesis thermal treatments should enable multi-metallic and alloy aerogels for a variety of applications requiring tunable material and application performance.

## 4. Materials and Methods

### 4.1. CNF–Alginate–Cobalt Aerogel Synthesis

Hydrogels were prepared using CNF (University of Maine, Process Development Center, Orono, ME, USA) with carboxymethyl concentration of 1.2 mmol/g and sodium alginate (Sigma Aldrich, Allentown, PA, USA). A 3% (*w*/*w*) sodium alginate solution and a 3% (*w*/*w*) CNF solution were prepared in deionized water. A 1:1 (*v*/*v*) ratio of the 3% (*w*/*w*) sodium alginate and 3% (*w*/*w*) CNF solutions was mixed to create a 3% (*w*/*w*) CNF–alginate solution with 1.5% alginate and 1.5% CNF by mass in the final solution. The mixture was placed on a stir plate and heated to 80 °C for 72 h and then cooled to ambient temperature. A volume of 1 mL of the 3% CNF–alginate solution was transferred into individual microfuge tubes and centrifuged at a relative centrifugal force (rcf) of 16,200× *g* for 5 min to compact the biopolymers and ensure CNF particle overlap. Following centrifugation, supernatant fluid was aspirated. The microfuge tubes with CNF–alginate gels were sealed and cooled at 4 °C for 24 h.

Salt solutions of 2 M cobalt (II) chloride (Sigma Aldrich, Allentown, PA, USA) and 2 M calcium chloride (Sigma Aldrich, Allentown, PA, USA) were prepared and mixed in a 1:1 (*v*/*v*) ratio to form a 2 M CoCl_2_–CaCl_2_ solution with 1 M CoCl_2_ and 1 M CaCl_2_ in the final solution. A volume of 1 mL of the CoCl_2_–CaCl_2_ solution was added to each microfuge tube with CNF–alginate gel followed by cooling at 4 °C for 48 h to allow the CoCl_2_–CaCl_2_ solution to diffuse through and equilibrate in the CNF–alginate gel. The 4 °C temperature was selected to enable CNF and alginate to adopt low-energy conformations, allowing stable ionic crosslinking of carboxyl groups. The gels were then sliced into approximately 3 mm thick, 7.5 mm diameter cylinders using a razor blade.

The gels were then placed in 10 mL 2 M sodium borohydride (Sigma Aldrich, Allentown, PA, USA) for 24 h to chemically reduce the Co^2+^ ions into cobalt metal. Following chemical reduction, gels were rinsed in 50 mL deionized water for 24 h. The gels were then transferred to 50% (*v*/*v*) deionized water and 50% (*v*/*v*) ethanol for 24 h, then 75% (*v*/*v*) ethanol and 25% (*v*/*v*) deionized water for 24 h, and then a 100% (*v*/*v*) ethanol solution for 24 h to facilitate complete solvent exchange. Gels were then supercritically dried with CO_2_ using a Leica EM CPD300 Automated Critical Point Dryer (Leica Biosystems, Buffalo Grove, IL, USA).

The supercritically dried gels were then pyrolyzed in a Nabertherm RSH 50/500/13/P480 tube furnace (Nabertherm, Livingston, NJ, USA). Prior to pyrolysis, gels were placed in the closed furnace system in ceramic crucibles for 30 min in flowing nitrogen gas at 1300 mL/min to ensure all O_2_ was removed. Pyrolysis was performed at 550 °C for one hour under N_2_ gas. Following pyrolysis, the thermal oxidation process was performed using the same furnace at 550 °C in air for 1 h.

### 4.2. Fourier Transform Infrared Spectroscopy (FTIR)

FTIR spectra were acquired with a PerkinElmer Frontier Optica FTIR spectrometer (PerkinElmer, Waltham, MA, USA) using attenuated total reflectance (ATR). Sample spectra wave number range was 4000–600 cm^−^^1^; 64 scans were collected with a resolution of 1 cm^−^^1^.

### 4.3. Scanning Electron Microscopy (SEM) and Energy Dispersive X-ray Spectroscopy (EDS)

SEM micrographs and EDS spectra were collected with a JEOL IT500HR SEM (JEOL USA, Peabody, MA, USA) and a Quattro S SEM (Thermo Fisher Scientific, Bothell, WA, USA). Samples were not sputter coated with metal prior to imaging to facilitate more accurate feature size analysis conducted with ImageJ [122].

### 4.4. X-ray Diffractometry (XRD)

XRD measurements were performed using a Malvern PANalytical Empyrean diffractometer (Malvern Panalytical, Almelo, Netherlands) equipped with a wide-angle X-ray scattering (WAXS) stage. Scans were collected at 45 kV and 40 mA with Cu_ka_ radiation, a 2θ step size of 0.0130°, and 20 s per step for diffraction angles performed from 5 to 90°. Thermal XRD measurements were collected using an Anton Paar TCU 1000N Temperature Control Unit. Samples were heated at 150 °C/min and held for 50 min prior to each scan. Thermal scans were collected from 300 to 800 °C in increments of 100 °C with a step size of 0.0130° 2θ, and 48 s per step in a scan range of 30–70° 2θ. XRD diffractogram analysis was performed with High Score software (PANalytical).

### 4.5. X-ray Photoelectron Spectroscopy (XPS)

The XPS spectra were collected using a K-Alpha XPS System (Thermo Fisher Scientific, Waltham, MA, USA) using a monochromated Al k_α_ X-ray source (1486.6 eV). Survey scans were acquired in constant analyzer energy mode with pass energy of 200 eV for survey scans and 50 eV for higher resolution scans. Charge compensation was achieved using a flood gun to provide low energy electrons. All spectra were collected with a 400 µm X-ray spot size. Thermo Avantage v5.9925 (Thermo Fisher Scientific, Waltham, MA, USA) was used for all spectral analysis, peak fitting, and atomic percentage calculations. Peaks were calibrated using the adventitious carbon peak at 284.8 eV. Samples were analyzed with no sputtering or cleaning treatments.

### 4.6. Thermal Gravimetric Analysis (TGA)

TGA was conducted on TA Instruments TGA 55 series (Thermal Instruments, New Castle, DE, USA). A high temperature platinum pan was used, with samples ranging from 4 to 8 mg. Samples were analyzed from ambient to 1000 °C with a 10 °C/min temperature ramp under both a 25 mL/min nitrogen flow and 25 mL/min air flow. The TA Instruments TRIOS software was used to determine the change in weight as a function of temperature.

### 4.7. Nitrogen Adsorption–Desorption Analysis

Nitrogen gas adsorption–desorption analysis was performed using a Micromeritics ASAP 2020 Plus (Micromeritics, Norcross, GA, USA) to determine surface area and pore size distribution according to IUPAC standards [100]. Nitrogen (−196 °C) was used as the test gas. Samples were vacuum degassed at 100 °C for 16 h prior to measurement. Specific surface area from gas adsorption was determined with Brunauer–Emmett–Teller (BET) analysis [101]. The Barrett–Joyner–Halenda (BJH) model [102] applied to volumetric desorption isotherms was used to calculate pore size distributions.

### 4.8. Mechanical Characterization

Aerogel compression testing was conducted with an Instron 68SC-1 Universal Tabletop Testing System (Instron, Norwwood, MA, USA). A 500 N load cell was used with strain rates of 1.3 mm/min up to a maximum 60% strain for supercritically dried samples. Due to the brittle nature of pyrolyzed and thermally oxidized samples, a 0.2 mm/min strain rate was used over a 60% strain range. Elastic moduli were estimated from the stress–strain elastic region within 0–0.1 strain. Elastic moduli for pyrolyzed and thermally oxidized samples were estimated from the stress-strain curves up to the point of initial fracture. Sample geometry was determined with Starrett 799A Digital Calipers (Starrett, Aukland, New Zealand).

### 4.9. Inductively Coupled Plasma Optical Emission Spectroscopy (ICP-OES)

ICP-OES measurements were collected using a Perkin Elmer Avio 500 ICP Optical Emission Spectrometer (Perkin Elmer, Shelton, CT, USA). Calibration curves were obtained in the 1–10 ppm range using the SCP Science quality control standard number 4.

### 4.10. Vibrating Sample Magnetometry (VSM)

VSM was performed using a MicroSense EZ7 vibrating sample magnetometer (MicroSense, Lowell, MA, USA). Hysteresis loops were measured in the −6000 to 6000 Oe range at a rate of 25 Oe/s. Data analysis was performed using MicroSense EasyVSM software and hysteresis loops were corrected for background signal, signal slope starting at 4000 Oe, signal offset, and sweep field lag.

### 4.11. Electrochemical Characterization

Electrochemical impedance spectroscopy (EIS) and cyclic voltammetry (CV) were performed using a Bio-Logic VMP-3 potentiostat (Bio-Logic, Knoxville, TN, USA). 1 M KOH solution was used as the supporting electrolyte in a 3-electrode cell, with an Ag/AgCl (3 M NaCl) reference electrode, and a 0.5 mm diameter platinum wire auxiliary electrode. Galvanostatic impedance spectra were collected with a current magnitude of 10 mV sine wave from 10^5^ to 10^−^^3^ Hz.

## Figures and Tables

**Table 1:** Nitrogen adsorption–desorption pore volume, BET, and BJH analysis values.

Sample	Cum. Pore Vol (cm^3^/g)	BET SSA (m^2^/g)	BJH SSA (m^2^/g)	BJH Avg Pore (nm)
Supercritically Dried	1.53	190	228	26.9
Pyrolyzed	0.69	111	93	30.0
Thermally Oxidized	0.02	3.4	2.7	22.8

**Table 2:** Magnetic measurements of the aerogel samples determined from VSM hysteresis loops.

Sample	M_s_ (emu/g_Co_)	M_r_ (emu/g_Co_)	Hc (Oe)
Supercritically Dried	1.72	0.1	46.36
Pyrolyzed	114.19	20.07	311.96
Thermally Oxidized	39.05	4.01	113.1

**Table 3:** Material characterization summary for supercritically dried, pyrolyzed, and thermally oxidized aerogels.

Sample	Supercritically Dried	Pyrolyzed	Thermally Oxidized
Predominant Cobalt Phase	Co(s)	Co(s)	Co_3_O_4_
Density (g/cm^3^)	0.16 ± 0.2	0.27 ± 0.08	0.24 ± 0.3
Fibril Diameter (nm)	22 ± 3	12 ± 2	77 ± 22
Atomic % Co:Ca	1.0:1.3	1.0:0.7	1.0:0.5
Cum. Pore Volume (cm^3^/g)	1.53	0.69	0.02
BET Specific Surface Area (m^2^/g)	190	111	3.4
BJH Specific Surface Area (m^2^/g)	228	93	2.7
BJH Avg Pore (nm)	26.9	30.0	22.8
M_s_ (emu/g_Co_)	1.72	114.19	39.05
M_r_ (emu/g_Co_)	0.1	20.07	4.01
Hc (Oe)	46.36	311.96	113.1
Elastic Moduli (MPa)	0.58 ± 0.54	1.1 ± 2.8	14.3 ± 2.9
EIS Specific Capacitance (F/g_Co_)	122	722	387

## Data Availability

The data presented in this study are openly available in article.

## Supplementary Materials

The following supporting information can be downloaded at https://www.mdpi.com/article/10.3390/gels9110893/s1, Figure S1—scanning electron microscopy and image analysis of spherical aggregates; Figure S2—scanning electron microscopy of thermally annealed samples; Figure S3—inductively coupled plasma chart; Table S1—ICP table; Figure S4—XPS survey scan; Figure S5—compressive stress–strain (low strain ranges); Figure S6—EIS/CV—individual samples; Figure S7—cyclic voltammetry of individual samples (50–1 mV/s range).
