# Supplementary material for: Cellulose Nanofiber–Alginate Biotemplated Cobalt Composite Multifunctional Aerogels for Energy Storage Electrodes
Source: Gels. 2023 Nov 11;9(11):893. doi: 10.3390/gels9110893 (PMC10671317; doi:10.3390/gels9110893)
Supplement: Supplementary file 1 [file gels-09-00893-s001.zip › gels-2662707-supplementary.pdf]

Supporting Information

Cellulose Nanofiber-Alginate Biotemplated Cobalt Composite Multifunctional Aerogels for Energy Storage

Felita W. Zhang [1], Paul D. Trackey [1], Vani Verma [1], Galen T. Mandes [1], Rosemary L. Calabro [1,2], Anthony W. Presot [1], Claire K. Tsay [1], Timothy J. Lawton [3], Alexa S. Zammit [1], Edward M. Tang [1], Andrew Q. Nguyen [1], Kennedy V. Munz [1], Enoch A. Nagelli [1,4], Stephen F. Bartolucci [2], Joshua A. Maurer [2] and F. John Burpo [1,4,*]

1 Department of Chemistry and Life Science, United States Military Academy, West Point, NY 10996, USA; felita.zhang@westpoint.edu (F.W.Z.); paul.trackey@westpoint.edu (P.D.T.); vani.verma.mil@army.mil (V.V.); galen.mandes@westpoint.edu (G.T.M.); rosemary.calabro@westpoint.edu (R.L.C.); anthony.presot@westpoint.edu (A.W.P.); claire.tsay@westpoint.edu (C.K.T.); alexa.zammit@westpoint.edu (A.S.Z.); edward.tang@westpoint.edu (E.M.T.); andrew.nguyen@westpoint.edu (A.Q.N.); kenndey.munz@westpoint.edu (K.V.M.); enoch.nagelli@westpoint.edu (E.A.N.)

2 U.S. Army Combat Capabilities Development Command-Armaments Center, Watervliet Arsenal, NY 12189, USA; stephen.f.bartolucci.civ@army.mil (S.F.B.); joshua.a.maurer4.civ@army.mil (J.A.M.)

3 U.S. Army Combat Capabilities Development Command-Soldier Center, Natick, MA 01760, USA; timothy.j.lawton4.civ@army.mil

4 Photonics Research Center, United States Military Academy, West Point, NY 10996, USA

* Correspondence: john.burpo@westpoint.edu; Tel.: +1-845-938-3900

Figures **Page**

S1. SEM (high resolution spherical aggregates; ImageJ analysis) 2

S2. SEM (thermal treatment) 3

S3. ICP chart 4

Table 1. Percent cobalt and calcium as determined from ICP-OES 5

S4. XPS survey scan 6

S5. Representative stress-strain curves at low strain ranges 7

S6. EIS/CV – individual samples (from Figure 10) 8

S7. Cyclic voltammetry of individual samples 9

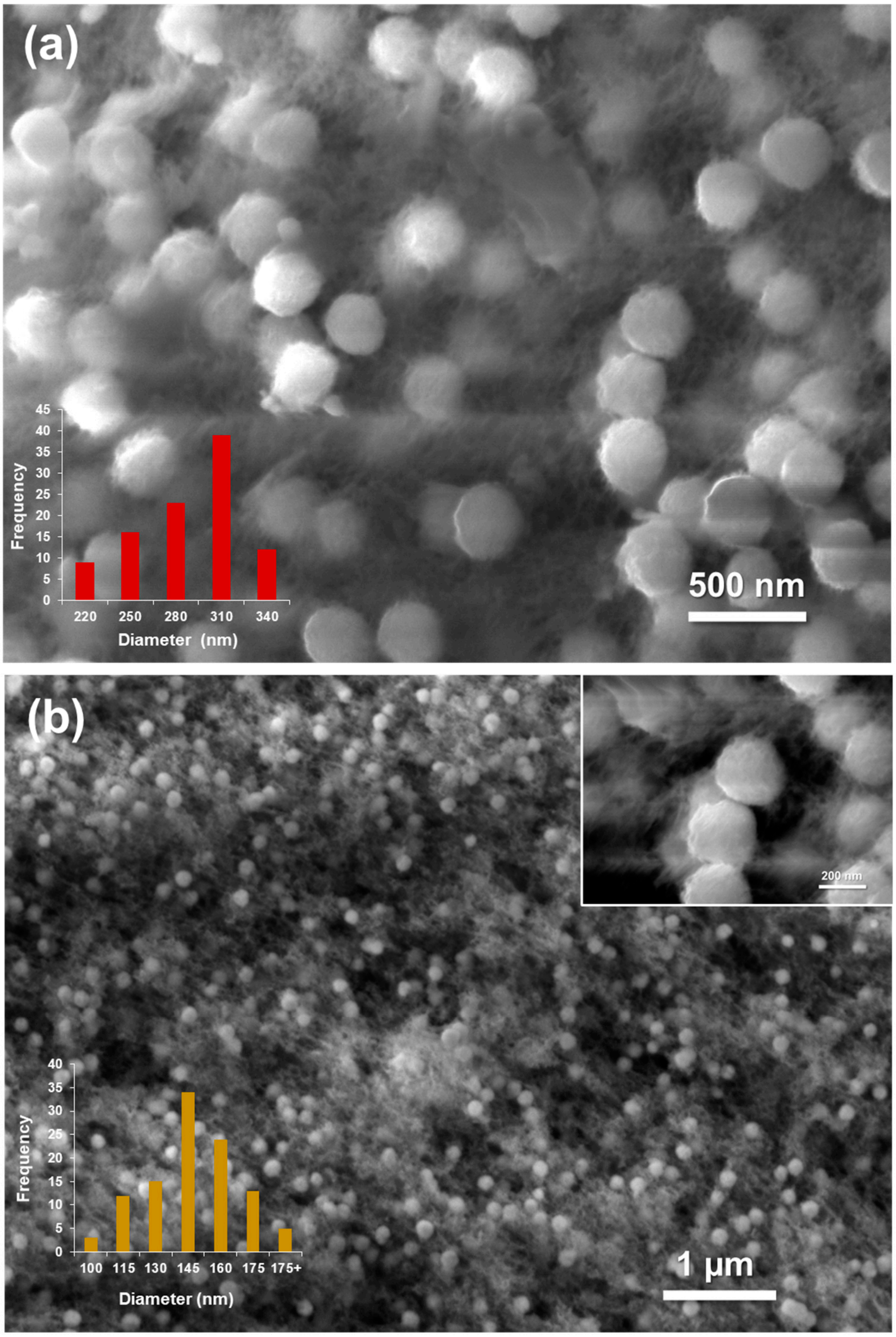

Figure S1. Scanning electron micrograph of **(a)** supercritically dried and **(b)** pyrolyzed aerogels focused on spherical aggregates with diameter distribution (inset). The average aggregate diameter is (a) 267 ± 53 nm and (b) 140 ± 22 nm.

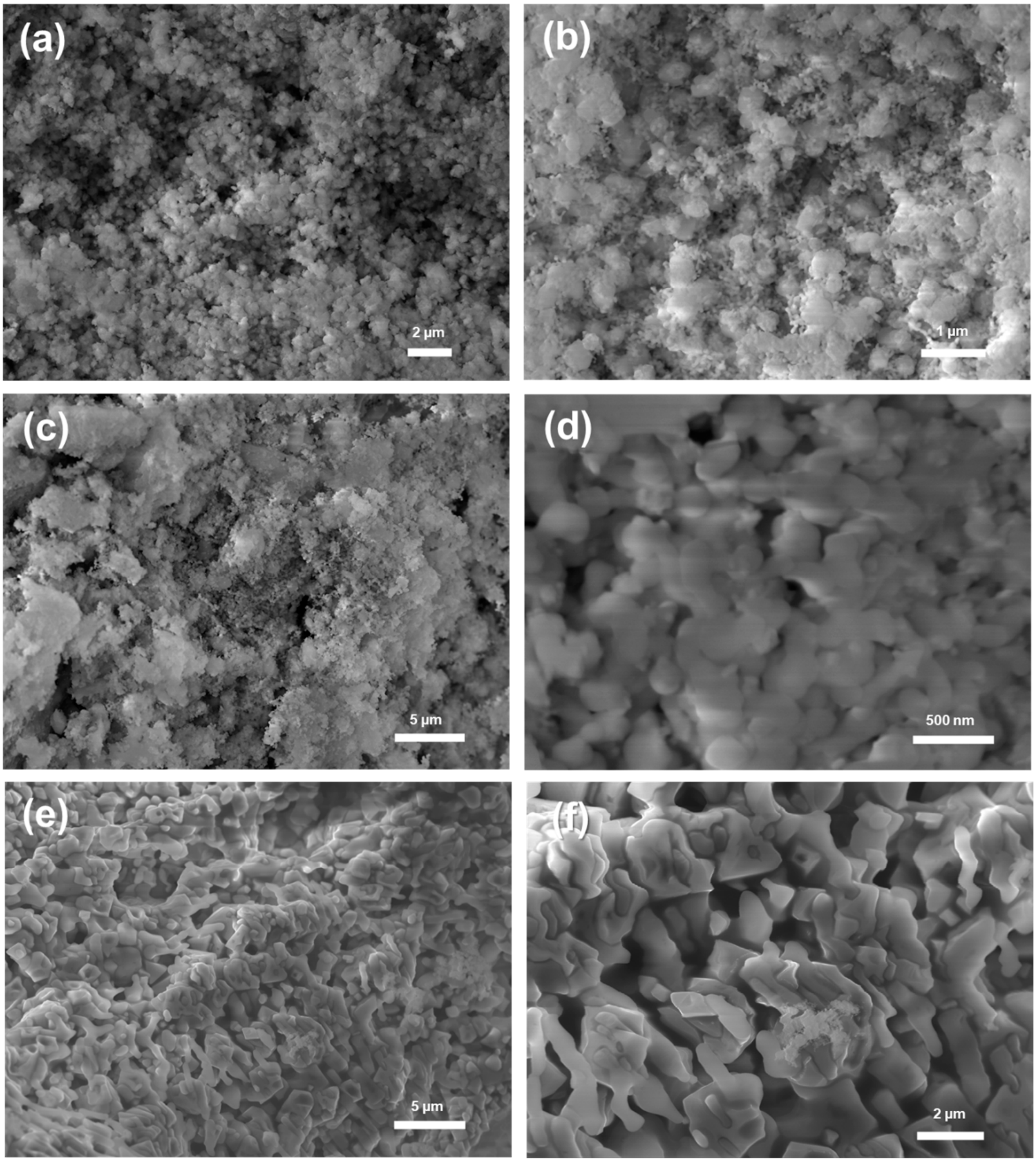

Figure S2. Scanning electron micrographs (SEM) of pyrolyzed aerogels thermally oxidized in air for **(a)-(b)** 550 °C for 1 h, **(c)-(d**) 600 °C for 30 min, and **(e)-(f)** 700 °C for 30 min.

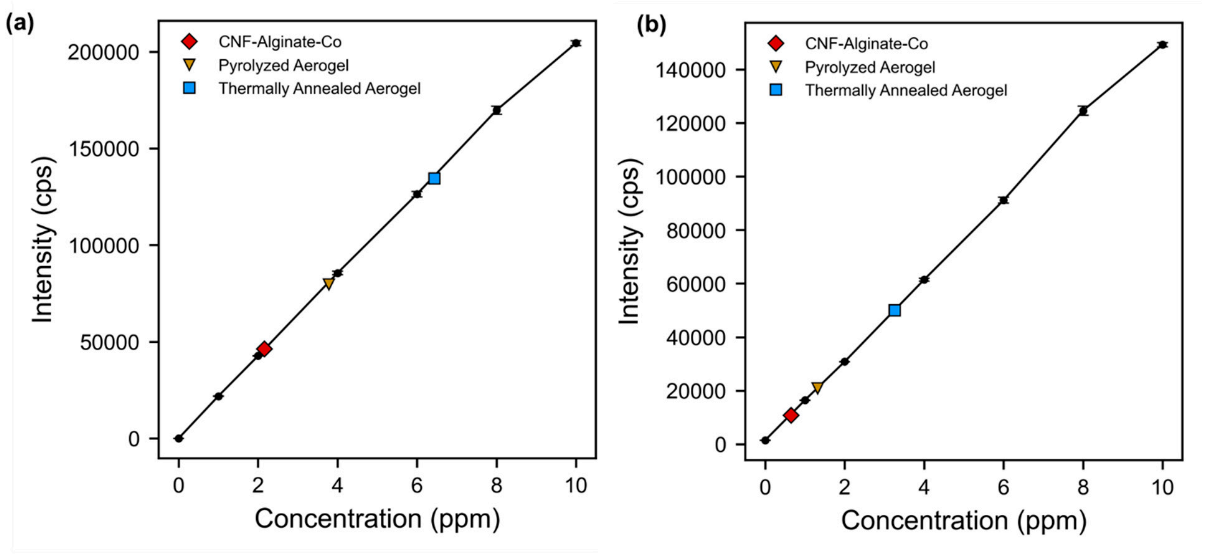

Figure S3. Inductively coupled plasma optical emission spectroscopy (ICP-OES) measurements for the cobalt **(a)** and calcium content **(b)** of the CNF-Alginate-Co samples at their various processing steps. The calibration curves (black dots) were measured using the SCP Science quality control standard number 4. The CNF-Alginate-Co (red diamonds), pyrolyzed aerogel (yellow triangles) and thermally annealed aerogel (blue squares) were digested in concentrated nitric acid and then diluted prior to analysis.

Table S1. Percent cobalt and calcium as determined from ICP-OES measurements.

Sample Conditions	Initial Mass (mg)	Digestion Conc. (mg / L)	Dilution Conc. (mg / L)	Co Conc (mg / L)	Ca Conc (mg / L)	% Co	% Ca
CNF-Alginate-Co	4.29	171.6	13.73	2.17	0.66	15.77	4.78
Pyrolyzed	3.84	153.6	12.29	3.79	1.32	30.81	10.76
Thermally Annealed	6.67	266.8	21.34	6.44	3.26	30.17	15.26

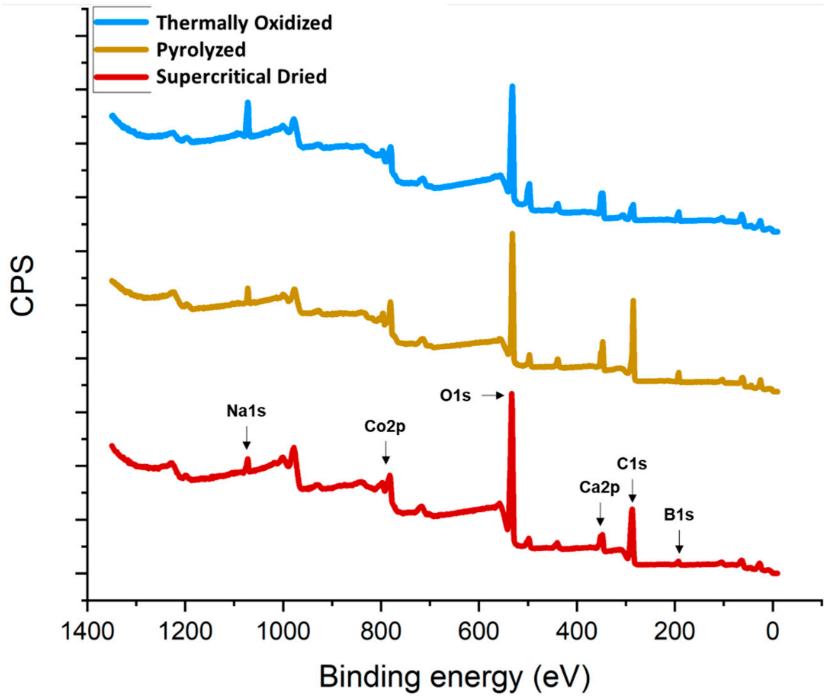

Figure S4. X-ray photoelectron spectra (XPS). Survey spectra for supercritically dried, pyrolyzed and thermally oxidized samples.

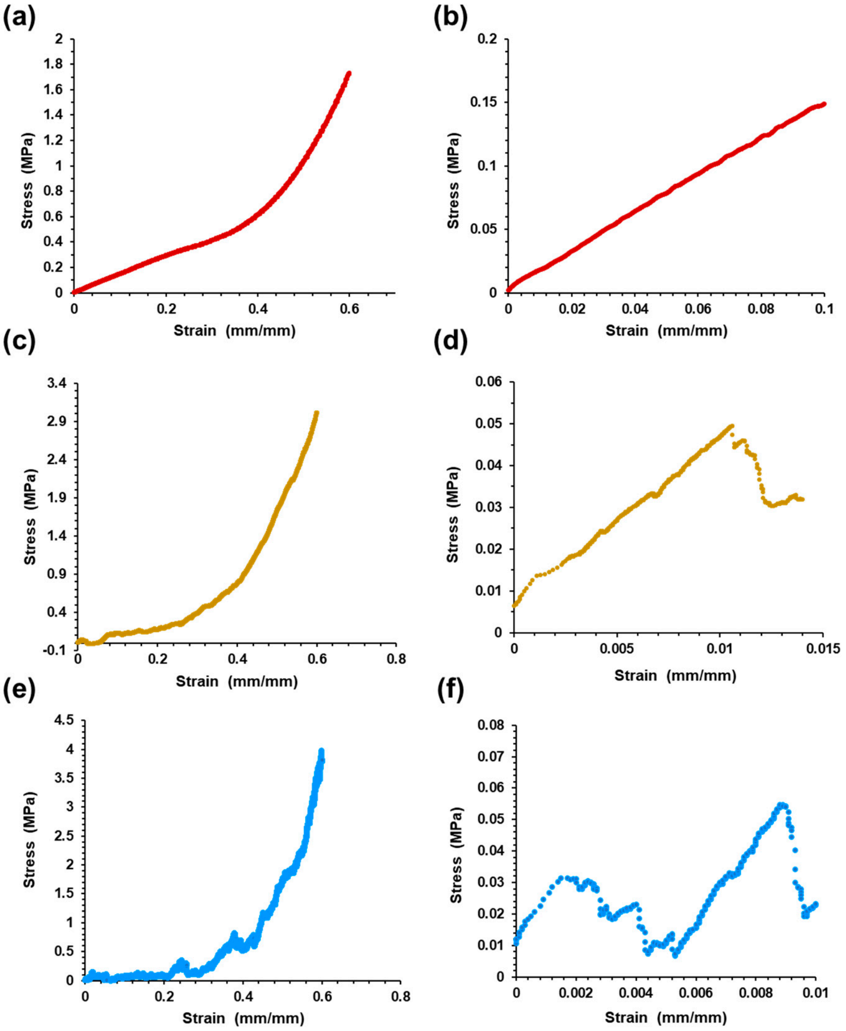

Figure S5. Representative aerogel compressive stress-strain curves and low strain range for **(a)-(b)** supercritically dried, **(c)-(d)** pyrolyzed, and **(e)-(f)** thermally oxidized aerogels.

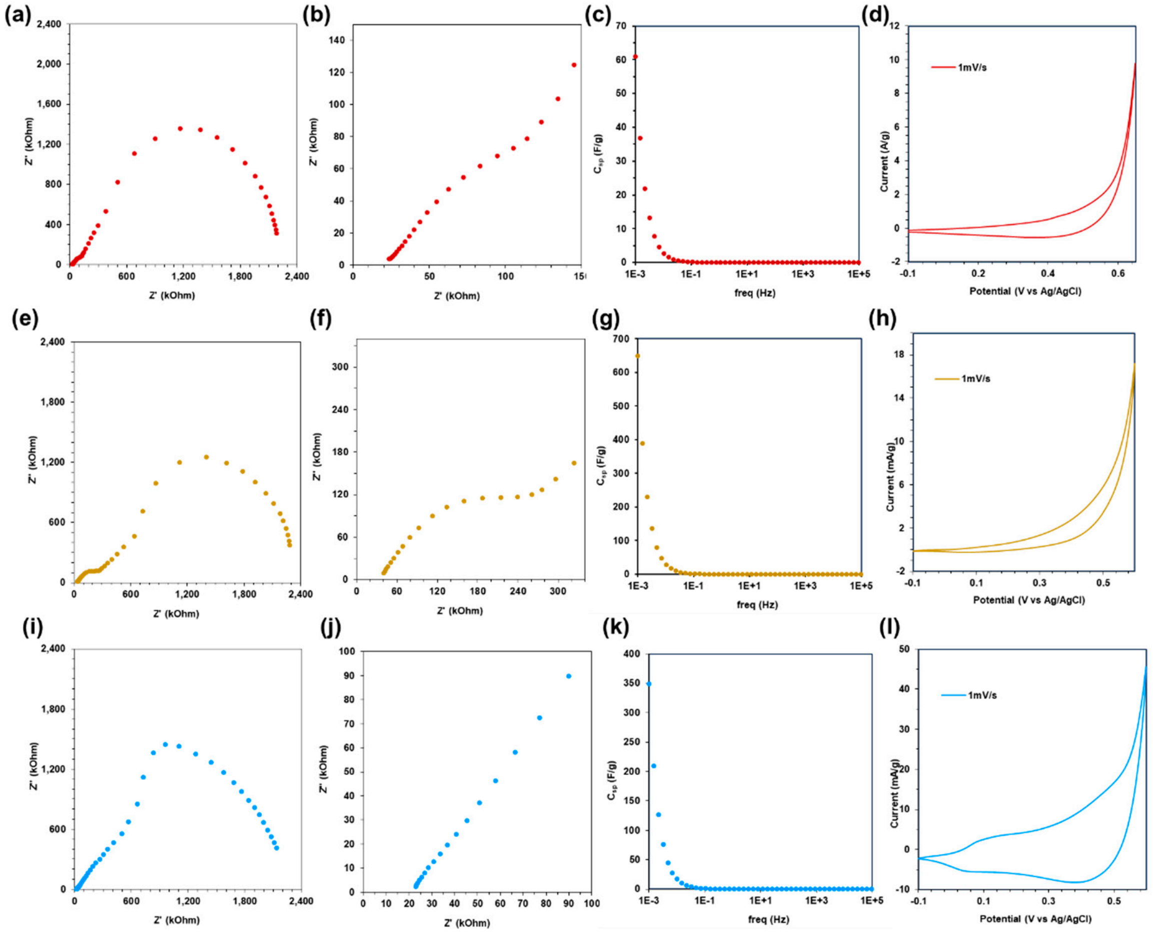

Figure S6. Electrochemical characterization in 1 M KOH. Electrochemical impedance spectroscopy (EIS) from 100 kHz to 1 mHz; high frequency impedance; specific capacitance (C_{sp}) vs frequency; and cyclic voltammetry at scan rate of 1 mV/s for **(a)-(d)** supercritically dried, **(e)-(h)** pyrolyzed, and **(i)-(l)** thermally oxidized aerogels.

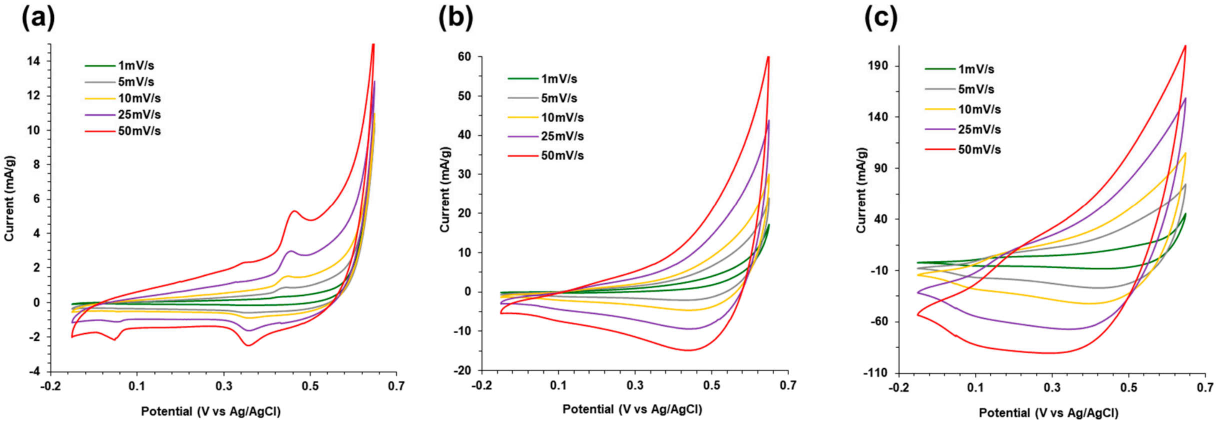

Figure S7. Cyclic voltammetry (CV) in 1 M KOH at scan rates of 50, 25, 10, 5, and 1 mV/s for **(a)** supercritically dried, **(b)** pyrolyzed, and **(c)** thermally oxidized aerogels.
